# Angle-dependent structural colors in a nanoscale-grating photonic crystal fabricated by reverse nanoimprint technology

**DOI:** 10.3762/bjnano.10.120

**Published:** 2019-06-11

**Authors:** Xu Zheng, Qing Wang, Jinjin Luan, Yao Li, Ning Wang, Rui Zhang

**Affiliations:** 1 Institue of NanoEngineering, College of Civil Engineering and Architecture, Shandong University of Science and Technology, 266590 Shandong, China

**Keywords:** observation angle, photonic crystal, reverse nanoimprint lithography, structural color, visualized sensor

## Abstract

The structural color of angle-sensitive photonic crystals has attracted great interest because of a possible application in visual sensors. The appearance of a photonic crystal is mainly influenced by the optical properties, structural parameters and the observation angle. In this work, an angle-sensitive photonic crystal with nanoscale gratings was fabricated through reverse nanoimprint lithography. The periodicity and the structural color were investigated through measuring reflection spectra. The structural color of the photonic crystal has a period of 90°. Distinctive colors spanning the entire visible spectrum can be seen when the crystal is rotated. In addition, there is a blue-shift of the peak wavelength when the observation angle is increased. An equation for the observed wavelength as a function of the observation angle is proposed.

## Introduction

Photonic crystals have been extensively investigated in recent years because of their potential application in visual sensors [1]. Much attention has been paid to photonic crystals the reflection wavelength of which changes with the observation angle [2]. The photonic crystals can exhibit different brilliant structural colors in the visible-light region. As period and depth of the optical structure of the photonic crystal are fixed, the structural color depends on the observation angle [3–4]. Studies show that photonic crystals with grating pattern are the favorable candidates for a variety of applications, not only due to their compact structures, simple fabrication process and excellent spectral characteristics, but also because reflection wavelength and observation angle are closely connected [5–7]. At present, nanoimprint lithography (NIL) and self-assembly are the two main methods to prepare photonic crystals with grating pattern [8–11]. And NIL is most often reported because it is inexpensive and easily applied [12–15].

The appearance of a photonic crystal is mainly influenced by the optical properties, structural parameters and the observation angle [16–19]. Duempelmann et al. fabricated asymmetric periodic nanostructures to explore the effects of the optical properties on the structural color [20]. Then, they used the photonic crystal as a strain sensor by mechanically changing the structural period to achieve the different structural colors. Koirala et al. investigated the transmission filtering characteristics of a grating film while changing structural parameters including the period of the grating [21]. Siddique et al. fabricated hierarchical photonic nanostructures to discuss the influence of structure patterns on the reflection wavelength [22]. Since optical properties and the structural parameters of photonic crystals are fixed after fabrication, the researchers explored the dependence of the structural color on the observation angle. Yetisen et al. explored the influence of reflection angle on structural color based on the angle-sensitive photonic crystal [23]. Similarly, Zheng et al. also investigated the reflection wavelength while varying the incident angle on grating patterns [24]. The angle is one of the important factors affecting the structural color of photonic crystals. However, the angle parameters, such as horizontal and vertical observation angle, have not been investigated for a grating photonic crystal. The objective of this research is to provide an empirical relationship between observation angles and the structural color of an angle-sensitive nanoscale photonic crystal.

In this paper, an angle-sensitive nanoscale grating photonic crystal fabricated through reverse nanoimprint lithography is investigated. The photonic crystal exhibited a wide range color spectrum from red to blue. Periodicity and angle-dependence of the reflection wavelengths are explored through measuring reflectance spectra. Based on the experimental results, a function for reflection wavelength and observation angle is proposed. These results are useful for a possible application as visual sensor.

## Results and Discussion

**Figure 1:**
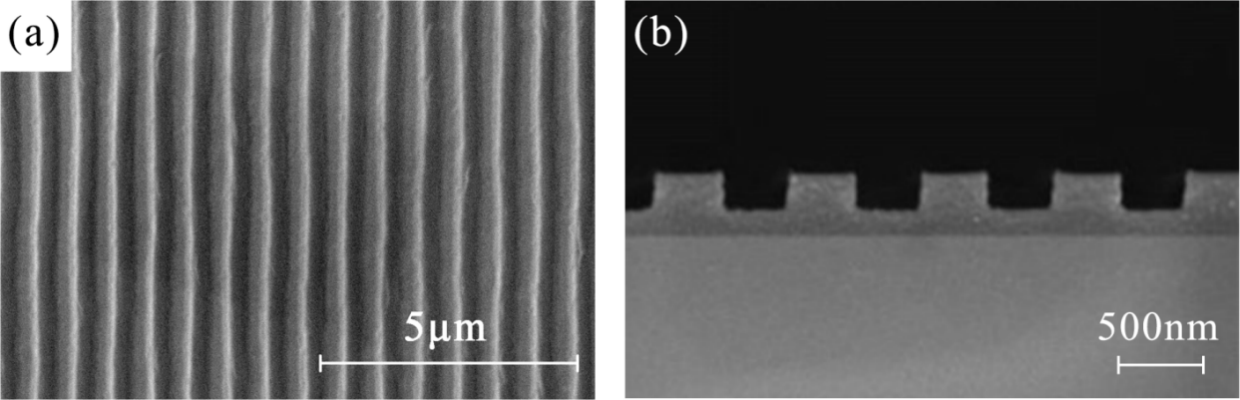
The SEM images of the fabricated photonic crystal film. (a) Top view, (b) cross-sectional view.

Figure 1 shows scanning electron microscopy (SEM) images of the photonic crystal with nanoscale grating pattern. The top-view SEM image of the fabricated photonic crystal film in Figure 1a clearly shows the grating structure with a period of 750 nm. The cross-sectional view is shown in Figure 1b. It can be seen that the mould is completely filled and the grating pattern has a height of 170 nm. The results show that the mould patterns were perfectly transferred to the film surface through reverse NIL.

### Periodicity of photonic crystals

**Figure 2:**
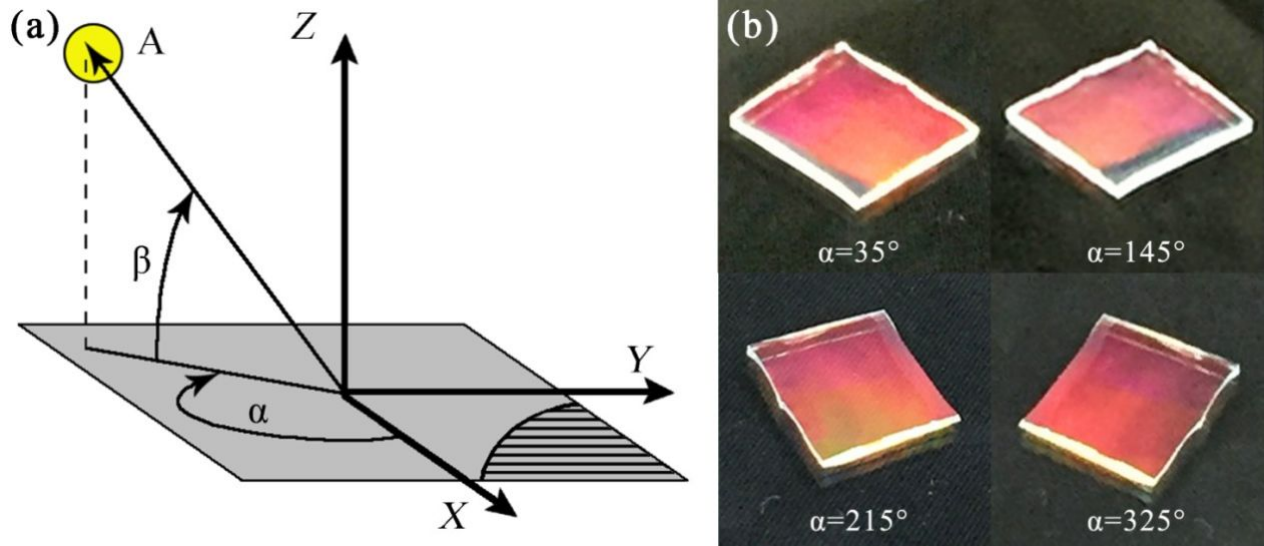
(a) Schematic diagram of the observation angles. (b) Photonic crystal structural color for horizontal observation angles of 35°, 145°, 215° and 325° under a vertical observation angle of 45°.

The structural color of angle-sensitive photonic crystals is related to the observation angle. Therefore, the effects of the horizontal and the vertical observation angle on the measured structural color of photonic crystals will be explored. Figure 2a is the schematic diagram of the observation angles. O is the center point of the photonic crystal, the *x*-axis is vertical to the direction of the grating, the *y*-axis is parallel to the direction of the grating, A is the observation point, α is the horizontal observation angle, and β is the vertical observation angle. The lower right corner of Figure 2a shows he direction of the grating.

Figure 2b shows the structural color for horizontal observation angles of 35°, 145°, 215° and 325° when the vertical observation angle is 45°. The structural color is the same for different observation angles. The reason for this phenomenon is that the horizontal angle between the observation point A and the grating pattern is constant with 55°. The structural color of the photonic crystal has a four-fold periodicity as the horizontal observation angle changes. The structural color of the photonic crystal in a period from 0° to 90° will be described below.

### Effects of horizontal observation angle on structural color

**Figure 3:**
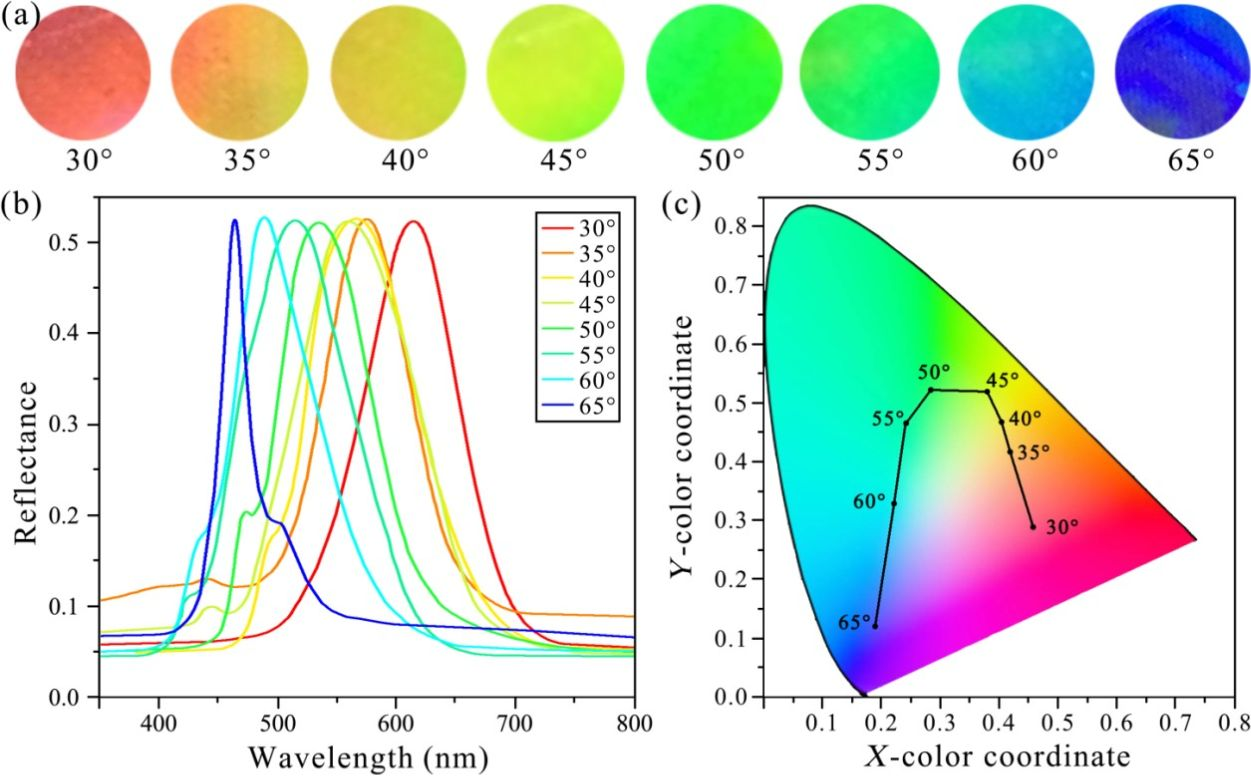
The relationship between horizontal observation angle and structural colors of the photonic crystal. (a) Color change of the photonic crystal as the horizontal observation angle is increasing. (b) Measured reflected spectra of white light for the photonic crystal under horizontal observation angles from 30° to 65°. (c) Measured peaks of the reflected spectra in the CIE 1931 *xy* chromaticity diagram.

Figure 3a shows an obvious color change from red to dark blue with increasing horizontal observation angle under the vertical observation angle of 45°. Figure 3b shows the reflection wavelength of the photonic crystal as a function of the horizontal observation angle measured with a fiber spectrometer. Generally, the maximum reflectance is larger than 50%. When the horizontal observation angle changes from 30° to 65°, the reflection wavelength peak changes from 615 to 465 nm. For a better illustration, Figure 3c shows a CIE color plot (the CIE 1931 *xy* chromaticity diagram) containing the reflection wavelength peaks. The chromaticity diagram shows that there is a blue-shift of the color with increasing horizontal observation angle. It can be seen from the graph that the color of the grating is distinctly different under different horizontal observation angles. The color shift is visible to the naked eye throughout most of the measurement cycle.

### Effects of vertical observation angle on structural color

**Figure 4:**
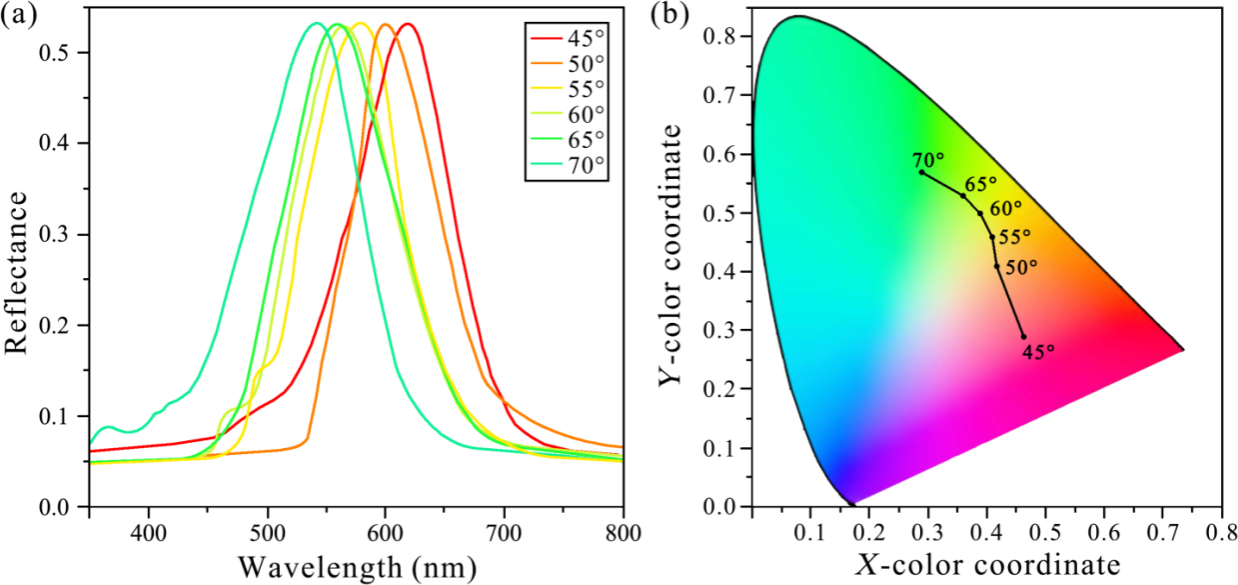
The relationship between vertical observation angle and structural color of the photonic crystal. (a) Measured reflected spectra of white light for horizontal observation angles from 45° to 70°. (b) Measured peaks of the reflected spectra in the CIE 1931 *xy* chromaticity diagram.

The fiber spectrometer was also used to measure the reflection wavelength for changing vertical observation angles. As shown in Figure 4a, when the vertical observation angle increases from 45° to 70°, under a constant horizontal observation angle of 30°, the peak wavelength originating from the structure shifts from 620 to 542 nm. The reflection wavelength peak locations in the CIE 1931 *xy* chromaticity diagram are presented in Figure 4b, which shows that the color is blue-shifted after the increase of the vertical observation angle. The color range of the vertical observation angle is much smaller than that of the horizontal observation angle.

### Relating observation angle and reflection wavelength peak

**Figure 5:**
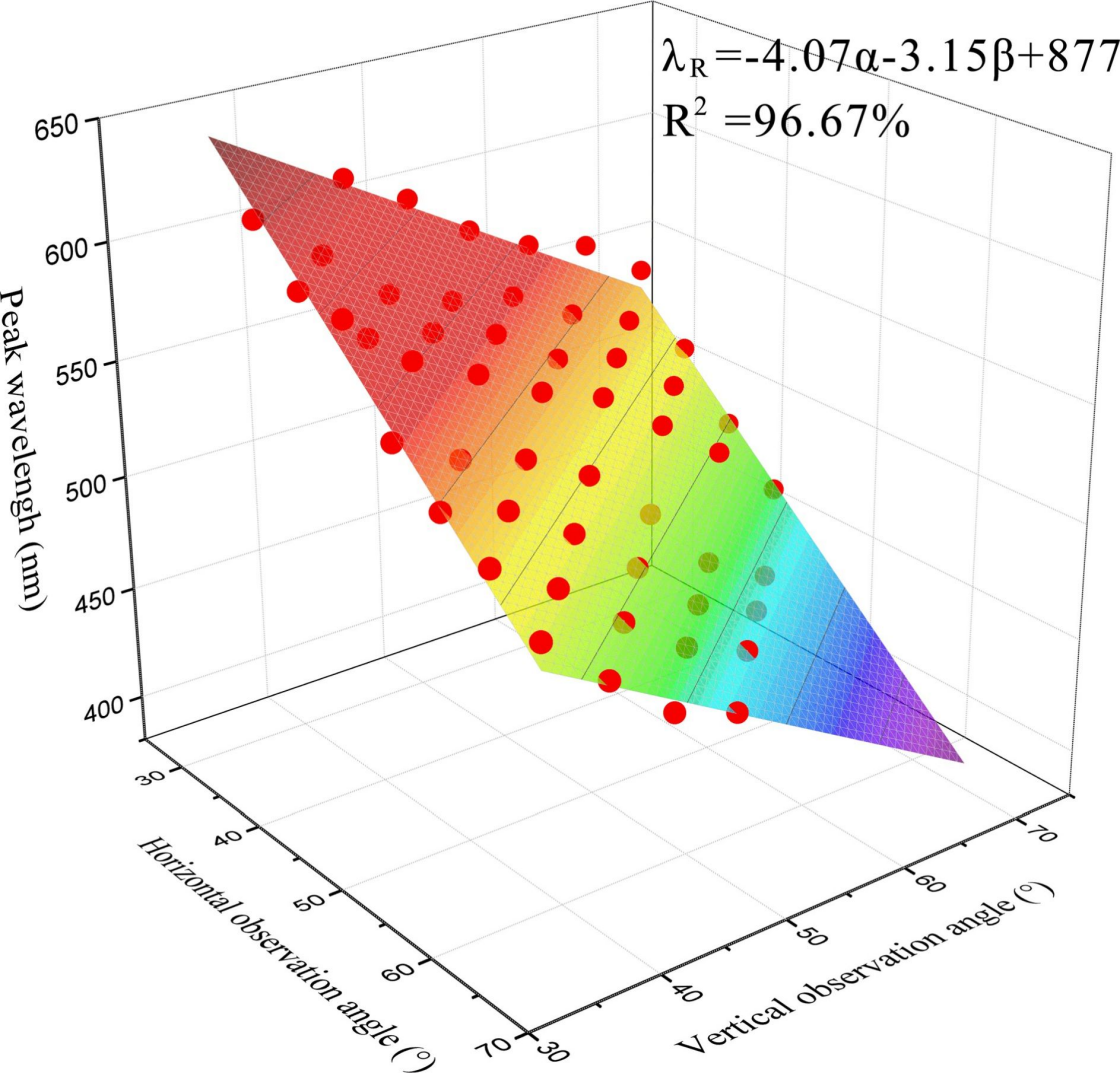
The three-dimensional function of the reflection wavelength peak and observation angle.

**Figure 6:**
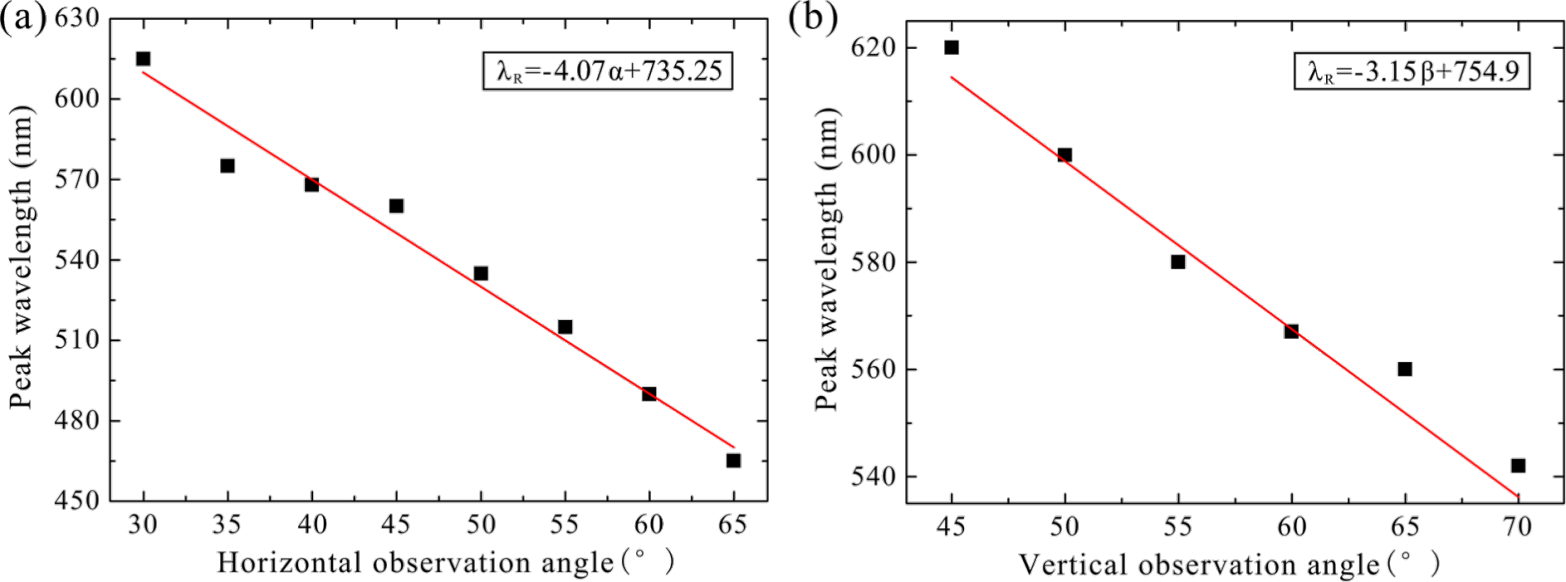
(a) Reflection wavelength peak as a function of the horizontal observation angle under a vertical observation angle of 45°. (b) Reflection wavelength peak as a function of the vertical observation angle under a horizontal observation angle of 30°.

A three-dimensional function of observation angle and reflection wavelength peak was obtained from Figure 5 through the analysis of reflection wavelength peak data. From the fit of the surface data, the following function relating the reflection wavelength peak to the horizontal and vertical observation angles is obtained:

[1] $$\lambda_{\text{R}}=-4.07\alpha -3.15\beta +877\;,$$

where the λ_R_ is the reflection wavelength peak in nanometers. The coefficient of correlation, *R*^2^, is 96.67%. When the structural parameters of the pattern and the sample material are fixed, the structural color is only related to the observation angles. It can be seen from the function that the reflection wavelength peak is negatively correlated with the observation angle. In Figure 6, the influence of a single factor is examined when the other factor is fixed. For a vertical observation angle of 45° or a horizontal observation angle of 30°, when substituted into Equation 1, the functions relating the reflection wavelength peak to the observation angles are:

[2] $$\lambda_{\text{R}}=-4.07\alpha +735.25\;,$$

[3] $$\lambda_{\text{R}}=-3.15\beta +754.9\;.$$

It can be clearly seen that the reflection wavelength peak is negatively correlated with these two factors. The scatter points in Figure 6 are the measured experimental data, and the red lines are the single-variable functions of Equation 2 and Equation 3. This relationship is confirmed by previous research results [24]. As shown in Figure 6, the photonic crystal produces rich color changes when a single observation angle is varied.

## Conclusion

An angle-sensitive photonic crystal with grating pattern was fabricated through reverse NIL to investigate the effects of horizontal and vertical observation angles on its structural color. The results of the structural color indicated that the photonic crystal had a period of 90°, which was due to the periodicity of the angle between the observation point and the grating pattern. Distinctive colors from red to blue were generated by changing the observation angle. The reflection wavelength peak changed from 615 to 465 nm as the horizontal observation angle increased from 30° to 65°, and the main wavelength was blue-shifted with increasing vertical observation angle. A function was fitted to the data to relate the reflection wavelength to both observation angles. These results are important for the investigation of the structural color of the angle-sensitive photonic crystal, as well as for applications of visual sensors.

## Experimental

A polycarbonate (PC) master mould with period of 750 nm and height of 170 nm was fabricated through NIL. The polydimethylsiloxane (PDMS) and cross-linker were obtained from Sylgard 184 (Dow Corning, USA).

The photonic crystal film with grating patterns was fabricated by PDMS. The grating patterns were replicated through reverse NIL (NIL-150, China) by the patterned PC master mould with a 10:1 (weight ratio) mixture of elastomer and cross-linker. The fabrication of PDMS photonic crystal films could be divided into four steps: (1) the prepolymer solution was firstly poured onto the PC mould after degassing; (2) the PDMS film was fabricated through spin-coating at a speed of 500 rpm; (3) the photonic crystal was fabricated by reverse imprinting with the parameters of 0.5 MPa and 80 °C for 4 h to complete filling and cross-linking; (4) the photonic crystal was peeled-off from the PC mould.

The surface structures of photonic crystal were obtained by SEM (Nova Nano SEM450, USA). The spectroscopic analyses were performed with an Ocean Optics fiber-optic spectrophotometer. The data analysis and fitting were performed by using Origin software.
